# The Combined Influence of Hydrostatic Pressure and Temperature on Nonlinear Optical Properties of GaAs/Ga_0.7_Al_0.3_As Morse Quantum Well in the Presence of an Applied Magnetic Field

**DOI:** 10.3390/ma11050668

**Published:** 2018-04-25

**Authors:** Zhi-Hai Zhang, Jian-Hui Yuan, Kang-Xian Guo

**Affiliations:** 1 College of Physics and Electronic Engineering, Yancheng Teachers University, Yancheng 224007, China; 2 Department of Physics, Guangxi medical university, Nanning, Guangxi 530021, China; 3 Department of Physics, College of Physics and Electronic Engineering, Guangzhou University, Guangzhou 510006, China; kxguo@gzhu.edu.cn

**Keywords:** optical properties, quantum well, hydrostatic pressure, temperature, magnetic field

## Abstract

Studies aimed at understanding the nonlinear optical (NLO) properties of GaAs/Ga_0.7_Al_0.3_As morse quantum well (QW) have focused on the intersubband optical absorption coefficients (OACs) and refractive index changes (RICs). These studies have taken two complimentary approaches: (1) The compact-density-matrix approach and iterative method have been used to obtain the expressions of OACs and RICs in morse QW. (2) Finite difference techniques have been used to obtain energy eigenvalues and their corresponding eigenfunctions of GaAs/Ga_0.7_Al_0.3_As morse QW under an applied magnetic field, hydrostatic pressure, and temperature. Our results show that the hydrostatic pressure and magnetic field have a significant influence on the position and the magnitude of the resonant peaks of the nonlinear OACs and RICs. Simultaneously, a saturation case is observed on the total absorption spectrum, which is modulated by the hydrostatic pressure and magnetic field. Physical reasons have been analyzed in depth.

## 1. Introduction

In recent years, many physics researchers have placed much efforts in the nonlinear optical (NLO) properties of dimensionality semiconductor nanostructures in both theoretical and applied physics fields [1,2,3,4]. Moreover, the NLO properties arising out of intersubband transitions in dimensionality semiconductor nanostructures highlight important fundamental physics that are much more intense than those of bulk materials. The main reason is the quantum confinement of the charge carriers in dimensionality semiconductor nanostructures, which leads to the enhancement of the density of states at specific energies, the formation of discrete energy levels, and the drastic change in optical absorption spectra. Many novel NLO properties emerge in the low dimension of semiconductor structures. These properties can engender novel optical equipment [5,6].

Recently, the linear and NLO properties of dimensionality semiconductor nanostructures have become an essential and exciting field in physics. Optical absorption coefficients (OACs) and refractive index changes (RICs) in quantum well (QW) systems have attracted much attention [7,8,9,10]. It is well known that the linear and NLO properties can be affected by applied magnetic fields, applied electric fields, temperature and their combinations, which can tune the band gap of dimensionality semiconductor nanostructures; thereby, the change in optical properties may be applied to opto-electronic and electro devices. U. Yesilgul and F. Ungan et al. have investigated the NLO properties of double semi-V-shaped QWs under temperature, applied magnetic field, and hydrostatic pressure, finding that all these external stimuli can considerably modulate the optical properties [11]. H. Sari et al. have theoretically studied the effects of the non-resonant intense laser field, electric and magnetic fields on the NLO properties in n-type double δ-doped GaAs QW. The results show that the magnitudes and positions of resonant peaks of the total OACs and RICs show a considerable sensitivity to the strength of applied external fields [12]. Yuan et al. studied the second harmonic generation susceptibility in asymmetrical and symmetrical Gaussian potential QW with external electric fields. The results show that the second harmonic generation susceptibility depends dramatically on the applied electric field and the structural parameters [13].

Moreover, the morse QW system has been realized by coupling QWs of different thicknesses or by compositional grading of the energy bandgap, which has a small well depth and large asymmetry, indicating the existence of significant NLO properties. Some authors have researched the NLO properties in the morse QW system. Yu and coworkers have studied the optical rectification considering the polaron effect and OACs in morse QWs [14] Later on, R.L. Restrepo and E. Kasapoglu et al. studied the second and third harmonic generation associated with infrared transitions in morse QW with applied magnetic and electric fields [15]. S. Sakiroglu studied linear and nonlinear OACs and RICs in morse QW in terms of applied electric field [16]. However, a systematic study of the second harmonic generation susceptibility of optical nonlinearity in this system under the combined influence of hydrostatic pressure and temperature is still lacking. Therefore, research in this field is still needed, both theoretically and for practical applications. It is worth noting that, due to the external factor, it is not possible in general to find analytical solutions using common standard procedures. To overcome this difficulty, we perform a finite-difference calculation of the electronic state.

In the present work, we present a numerical study of nonlinear OACs and RICs in GaAs/AlGaAs morse QW. Additionally, the influence of hydrostatic pressure and temperature on nonlinear OACs and RICs has been taken into account. The Hamiltonian system and the relevant wave functions and energy levels are briefly described in Section 2. Additionally, the analytical expression of the nonlinear OACs and RICs are presented. The numerical results and discussions for GaAs/AlGaAs morse QW are given in Section 3. A brief summary is given in Section 4.

## 2. Theory

The GaAs/Ga_0.7_Al_0.3_As morse QW is systematically studied with the external magnetic field, under the effects of the hydrostatic pressure and temperature. The magnetic field is in-plane-oriented, taken along the confinement direction, B=(B,0,0), and the Landau gauge $A\left(r\right)=Bz\hat{y}$ is used. The Hamiltonian system in the effective mass approximation is given by [14,15,16]

(1) $$\left[-\frac{\hbar }{2}\frac{d}{d_{z}}\left[\frac{1}{m^{*}\left(P,T\right)}\frac{d}{d_{z}}\right]+V\left(z,P,T\right)+\frac{e^{2}B^{2}}{2m^{*}\left(P,T\right)c^{2}}\right]\varphi \left(z\right)=E\varphi \left(z\right)$$

where *z* represents the confinement direction of the QW, *T* is the temperature, $m^{*}\left(P,T\right)$ is the pressure (*P*)- and temperature (*T*)-dependent electron effective mass in the well region, given by the expression [11,17,18,19]

(2) $$\frac{m_{0}}{m^{*}\left(P,T\right)}=1+E_{p}^{\Gamma }\left[\frac{2}{E_{g}^{\Gamma }\left(P,T\right)}+\frac{1}{E_{g}^{\Gamma }\left(P,T\right)+\Delta_{0}}\right]$$

where $m_{0}$ is the free electron mass, $E_{p}^{\Gamma }=7.51$ eV is the energy related to the momentum matrix element, $\Delta_{0}=0.341$ eV is the spin-orbit splitting, and $E_{g}^{\Gamma }\left(P,T\right)=E_{g}^{\Gamma }\left(0,T\right)+bP+cP^{2}$ is the variation of the energy gap at the Γ-point for a GaAs semiconductor with a hydrostatic pressure and temperature as follows: $E_{g}^{\Gamma }\left(0,T\right)=\left[1.519-\left(5.405\times 10^{-4}T^{2}\right)/\left(T+204\right)\right]$ eV, $b=1.26\times 10^{-2}$ eV/kbar, and $c=-3.77\times 10^{-5}$ eV/kbar^2^.

**Figure 1:**
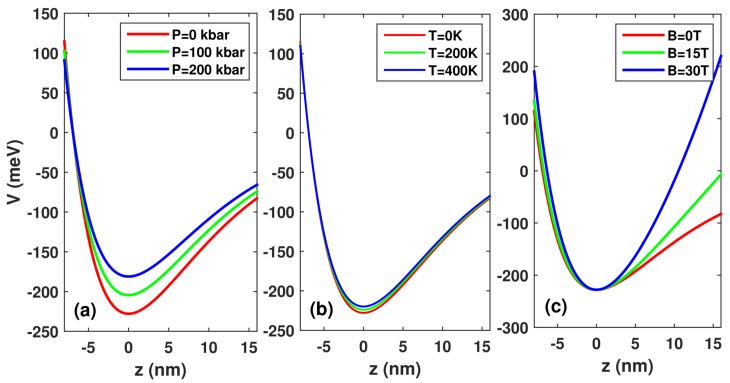
The variation in the confinement potential profile of morse quantum wells (QWs) for three different hydrostatic pressure *P* (**a**), temperature *T* (**b**), and applied magnetic field *B* (**c**) values.

The morse confinement potential V(z,x,P,T) is given by

(3) $$V\left(z,P,T\right)=V_{0}\left(P,T\right)\left(e^{-2\gamma z}-2e^{-\gamma z}\right)$$

where $V_{0}\left(P,T\right)=Q_{c}\Delta E_{g}^{\Gamma }\left(X,P,T\right)$ with $\Delta E_{g}^{\Gamma }\left(X,P,T\right)=\Delta E_{g}^{\Gamma }\left(X\right)+PD\left(X\right)+G\left(X\right)T$, $\Delta E_{g}^{\Gamma }\left(X\right)=\left(1.55X+0.37X^{2}\right)$ eV is the variation of the gap difference, $D\left(X\right)=[-\left(1.3\times 10^{-3}\right)X]$ eV/kbar, and $G\left(X\right)=[-\left(1.11\times 10^{-4}\right)X]$ eV/K. $Q_{c}=0.6$ is the conduction band offset parameter, and *X* is the aluminum concentration, which took a value of 0.3 in this paper. Note that the zero-potential is chosen at the right-hand barrier potential (see Figure 1).

External perturbations (hydrostatic pressure, temperature, and magnetic field) can modify the symmetry of the system, and the applied field affects the optical properties and becomes important in view of fundamental physics and device applications. However, due to the presence of the external perturbations in the Hamiltonian, it is impossible to find self-energy analytic eigenfunctions and eigenvalues that correspond to the exact solution of one electron confined in a low-dimensional quantum system. In this paper, the energy eigenvalues and eigenfunctions with an external magnetic field, temperature, and hydrostatic pressure are calculated using the finite difference method. Next, the compact-density-matrix method and the iterative procedure are used to derive the OACs and RICs in morse potential QWs. The analytical expressions of the linear and the third-order nonlinear susceptibilities for a two-level quantum system are given as follows [7,8,9,10]. First, for the linear term,

(4) $$\varepsilon_{0}\chi^{\left(1\right)}\left(\omega \right)=\frac{N{\left|M_{21}\right|}^{2}}{E_{21}-\hbar \omega -i\hbar \Gamma_{12}}.$$

For the third-order term,

(5) $$\begin{array}{cc} \varepsilon_{0}\chi^{\left(3\right)}\left(\omega \right)= & -\frac{N{\left|M_{21}\right|}^{2}}{E_{21}-\hbar \omega -i\hbar \Gamma_{12}}\\ & \times [\frac{4{\left|M_{21}\right|}^{2}}{{\left(E_{21}-\hbar \omega \right)}^{2}+{\left(\hbar \omega \right)}^{2}}\\ & -\frac{{\left(M_{22}-M_{11}\right)}^{2}}{\left(E_{21}-\hbar \omega -i\hbar \Gamma_{12}\right)}]. \end{array}$$

The susceptibility χ(ω) is related to the change in the refractive index as follows:

(6) $$\frac{\Delta n(\omega )}{n_{r}}=Re\frac{\chi (w)}{2n_{r}^{2}}$$

where $n_{r}$ is the refractive index. By using Equations (4)–(6), the changes in the linear and third-order nonlinear refractive indexes are obtained by

(7) $$\frac{\Delta n^{\left(1\right)}\left(\omega \right)}{n_{r}}=\frac{1}{2n_{r}^{2}\varepsilon_{0}}{\left|M_{21}\right|}^{2}N\left[\frac{E_{21}-\hbar \omega }{{\left(E_{21}-\hbar \omega \right)}^{2}+{\left(\hbar \Gamma_{12}\right)}^{2}}\right]$$

and

(8) $$\begin{array}{cc} \frac{\Delta n^{\left(3\right)}\left(\omega \right)}{n_{r}}= & -\frac{\mu c}{4n_{r}^{3}\varepsilon_{0}}{\left|M_{21}\right|}^{2}{\left[\frac{NI}{E_{21}-\hbar \omega }{\left(E_{21}-\hbar \omega \right)}^{2}+{\left(\hbar \Gamma_{12}\right)}^{2}\right]}^{2}\\ & \times [4\left(E_{21}-\hbar \omega \right){\left|M_{21}\right|}^{2}-\frac{{\left(M_{22}-M_{11}\right)}^{2}}{{\left(E_{21}\right)}^{2}+{\left(\hbar \Gamma_{12}\right)}^{2}}\{\left(E_{21}-\hbar \omega \right)\\ & \times \left[E_{21}\left(E_{21}-\hbar \omega \right)-{\left(\hbar \Gamma_{12}\right)}^{2}\right]-{\left(\hbar \Gamma_{12}\right)}^{2}\left(2E_{21}-\hbar \omega )\}\right] \end{array}$$

where μ is the permeability of the system, *N* is the carrier density in this system, $E_{ij}=E_{i}-E_{j}$ is the energy interval of two different electronic states, and $M_{ij}$ represents the matrix elements defined by $M_{ij}=|\langle \varphi_{i}\left|ex\right|\varphi_{j}\rangle |\left(i,j=1,2\right)$. *I* is the incident optical intensity and defined as

(9) $$I=2\sqrt{\frac{\varepsilon_{R}}{\mu }}{\left|E\left(\omega \right)\right|}^{2}=\frac{2n_{r}}{\mu c}{\left|E\left(\omega \right)\right|}^{2}$$

where *c* is the speed of light in free space. Therefore, the total refractive index change can be written as

(10) $$\frac{\Delta n(\omega )}{n_{r}}=\frac{\Delta n^{\left(1\right)}\left(\omega \right)}{n_{r}}+\frac{\Delta n^{\left(3\right)}\left(\omega \right)}{n_{r}}.$$

In addition, the susceptibility χ(ω) is related to the absorption coefficient α(ω) by

(11) $$\alpha^{\left(1\right)}\left(\omega \right)=\omega \sqrt{\frac{\mu }{\varepsilon_{R}}}\frac{{\left|M_{21}\right|}^{2}N\hbar \Gamma_{12}}{{\left(E_{21}-\hbar \omega \right)}^{2}+{\left(\hbar \Gamma_{12}\right)}^{2}}.$$

(12) $$\begin{array}{ccc} \alpha^{\left(3\right)}\left(\omega ,I\right) & = & -\omega \sqrt{\frac{\mu }{\varepsilon_{R}}}\left(\frac{I}{2\varepsilon_{0}n_{r}c}\right)\frac{{\left|M_{21}\right|}^{2}N\hbar \Gamma_{12}}{{\left[{\left(E_{21}-\hbar \omega \right)}^{2}+{\left(\hbar \Gamma_{12}\right)}^{2}\right]}^{2}}\left[4\right|M_{21}{|}^{2}\\ & - & \frac{|M_{22}-M_{11}{|}^{2}\left[3E_{21}^{2}-4E_{21}\hbar \omega +\hbar^{2}\left(\omega^{2}-\Gamma_{12}^{2}\right)\right]}{E_{21}^{2}+{\left(\hbar \Gamma_{12}\right)}^{2}}]. \end{array}$$

Therefore, the total absorption coefficient α(ω,I) is given by

(13) $$\alpha \left(\omega ,I\right)=\alpha^{\left(1\right)}\left(\omega \right)+\alpha^{\left(3\right)}\left(\omega \right).$$

## 3. Results and Discussion

In this section, we present our calculations for the OACs and RICs in a GaAs/Ga_0.7_Al_0.3_As morse QWs. The several constants we have used are $N=5\times 10^{16}$ ${\mathrm{cm}}^{-3}$, $n_{r}=3.2$, $T_{12}=0.14$ ps, and $\Gamma_{12}=1/T_{12}$.

**Figure 2:**
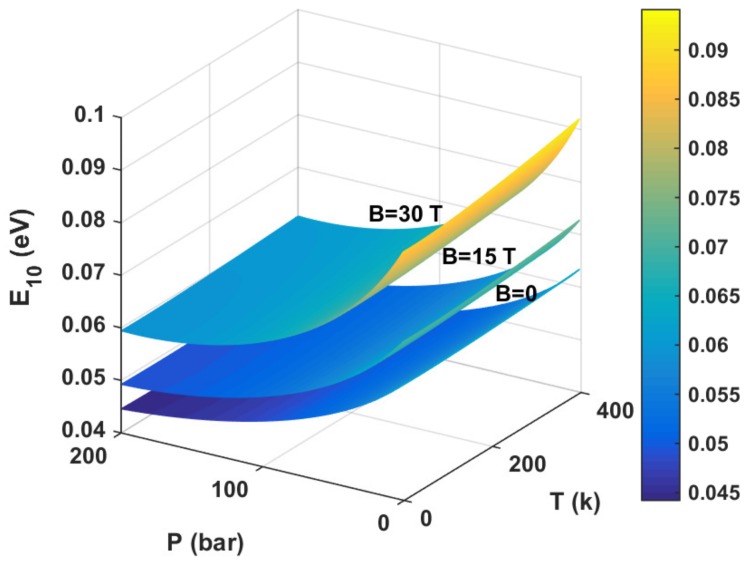
The energy difference $E_{10}$ of morse QWs are depicted as a function of hydrostatic pressure *P*, temperature *T*, and applied magnetic field *B*.

Figure 1 presents the changes in the confinement potential profile of morse QWs for (a) three different hydrostatic pressure *p* values with T=0,B=0, (b) three different temperature *T* values with P=0,B=0, (c) three different applied magnetic field *B* values with P=0,T=0. As seen from Figure 1a, as hydrostatic pressure *P* increases, the well width and the well depth decrease simultaneously. From Figure 1b, it is clear that, as the temperature *T* increases, the well width and well depth are slightly reduced. As can be seen in Figure 1c, as the applied magnetic field *B* increases, the well width decreases, which results in strong quantum confinement. In Figure 2, we demonstrate the energy difference between the ground state and the first excited state $E_{10}$ of the morse QW as a function of hydrostatic pressure *P*, temperature *T*, and magnetic field *B*. As seen, the energy difference $E_{10}$ always decreases with the increase in hydrostatic pressure *P*, which results in the the resonant peaks’ red-shift. Moreover, the energy difference $E_{10}$ always increases with the increase in applied magnetic field *B* and temperature *T*. Thus, we can easily understand the cause of the blue shift of the resonance of the photon.

**Figure 3:**
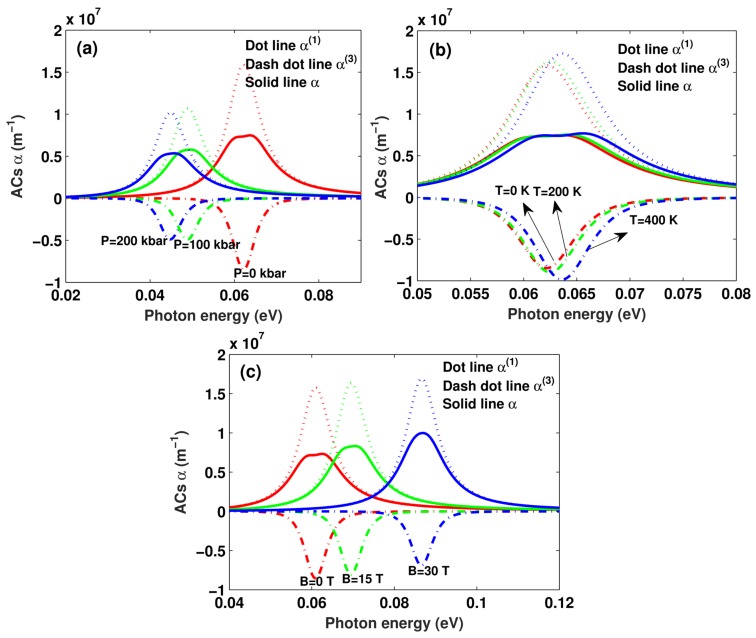
The OACs are depicted as a function of the incident photon energy for different hydrostatic pressure *P* (**a**), temperature *T* (**b**), and applied magnetic field *B* (**c**) with I=0.6 MW/cm^2^ and well width L=8 nm.

In Figure 3, we can observe the behavior of the OACs, calculated as a function of the photon energy. Several distinct values of (a) hydrostatic pressure (P = 0,100,200 kbar) with T=0,B=0, (b) temperature (T = 0,200,400 K) with P=0,B=0, and (c) magnetic field (B = 0,15,30 kbar) with P=0,T=0 have been taken into account for the incident optical intensity I=0.6 MW/cm^2^ and well width L=8 nm. We clearly observe that, in all curve, there is a resonant peak at a photon energy value, which corresponds to the energy difference between levels, since $\hbar \omega =E_{10}$. Total OACs α increase as linear absorption coefficient $\alpha^{\left(1\right)}$ increases(positive), but decrease as the third-order nonlinear absorption coefficient $\alpha^{\left(3\right)}$ increases(negative).

From Figure 3a, (1) We can see that, as an immediate effect of augmenting the magnitude of the hydrostatic pressure, the amplitude of the resonant peaks of the linear $\alpha^{\left(1\right)}$, third-order nonlinear $\alpha^{\left(3\right)}$, and total OACs α present a decrease in their heights or intensities, and the total OACs α is significantly split up into two peaks due to the strong absorption saturation. This is true with the exception of the case depicted in Figure 1a, the quantum confinement effect increases as well width decreases, which enlarges the amplitude of the resonant peaks of the linear $\alpha^{\left(1\right)}$, third-order nonlinear $\alpha^{\left(3\right)}$, total OACs α, and the energy interval. However, the effect of the well depth and the well width on the quantum confinement is opposite. The feature for the resonant peaks of the linear $\alpha^{\left(1\right)}$, third-order nonlinear $\alpha^{\left(3\right)}$, and total OACs α can be attributed to the complicated competitions between these two factors. Here, the change in well depth is the main factor influencing the resonant peaks of the linear $\alpha^{\left(1\right)}$, third-order nonlinear $\alpha^{\left(3\right)}$, and total OACs α, which causes the above results (see Figure 3a). (2) A very important feature of the figure is that the resonant peaks of the linear $\alpha^{\left(1\right)}$, third-order nonlinear $\alpha^{\left(3\right)}$, and total OACs α exhibit an obvious red-shift with the increase in hydrostatic pressure *P*. This is in complete accordance with the results of Figure 2.

From Figure 3b, (1) We can clearly see that the amplitude of the resonant peaks of the linear, third-order nonlinear, and total OACs increases with increasing temperature *T*. The physical reason for the behavior is that, as the temperature *T* rises, the quantum confinement effect decreases. This behavior is in general agreement with Figure 1b, we find the variation in the well width plays a major role in the change in the amplitude of the resonant peaks of the linear $\alpha^{\left(1\right)}$, third-order nonlinear $\alpha^{\left(3\right)}$, total OACs α, and energy difference. When the well width decreases, the wave function associated with the electron is less spread and more localized. Therefore, the amplitude of the resonant peaks increases. (2) The resonant peaks’ position shift towards higher energy with increasing temperature *T* can also be seen. The main reason for this behavior is the increase in the quantum confinement with increasing temperature *T*, which results in the increase in the energy difference between electronic states $E_{10}$. Therefore, the resonance peak position shifts towards higher energies. It is seen that our results in Figure 2 and Figure 3b are in fairly good agreement. Additionally, it is obvious that the hydrostatic pressure *P* and temperature *T* have opposite effects on the amplitude and position of the resonant peaks of the linear $\alpha^{\left(1\right)}$, third-order nonlinear $\alpha^{\left(3\right)}$, and total OACs α.

From Figure 3c, it can be seen that the amplitude of the resonant peaks of linear term $\alpha^{\left(1\right)}$ increases as applied magnetic field *B* increases. The opposite behavior takes place in the amplitude of the resonant peaks of third-order nonlinear term $\alpha^{\left(3\right)}$. Total OACs α is the sum of the linear term $\alpha^{\left(1\right)}$ and the third-order nonlinear term $\alpha^{\left(3\right)}$ (see Equation (13)), which increase with the increase in applied magnetic field *B*. It can also be seen that the resonant peaks of the OACs move toward high energy regions (blue shift) as the applied magnetic field *B* increases. These behaviors can be related to the quantum confinement effect. This is consistent with the results in Figure 1c. The blue shift occurs due to the effects of the applied magnetic field *B* on the energy levels, whereas the enhancement is caused by the increment in energy gap between states of the morse QWs and the incremented magnetic field. This is consistent with the results in Figure 2.

**Figure 4:**
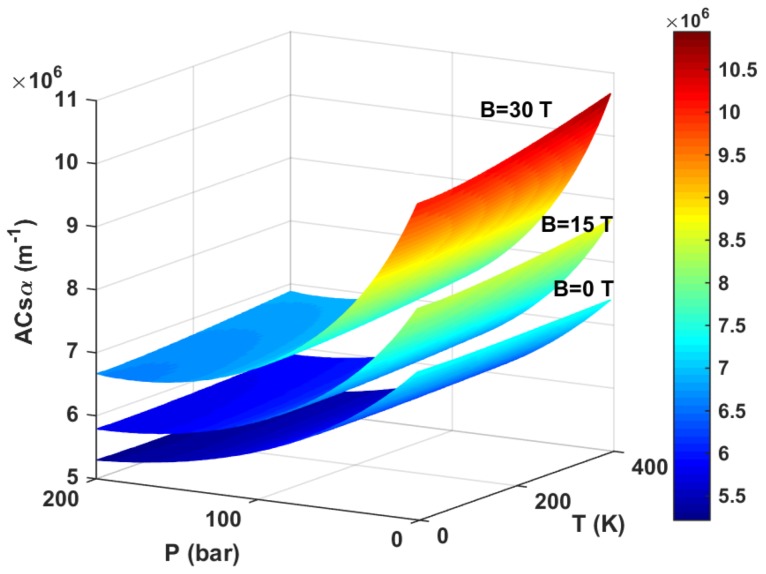
The resonance peak value of total OACs as a function of hydrostatic pressure *P*, temperature *T*, and applied magnetic field *B* with I=0.6 MW/cm^2^ and well width L=8 nm.

**Figure 5:**
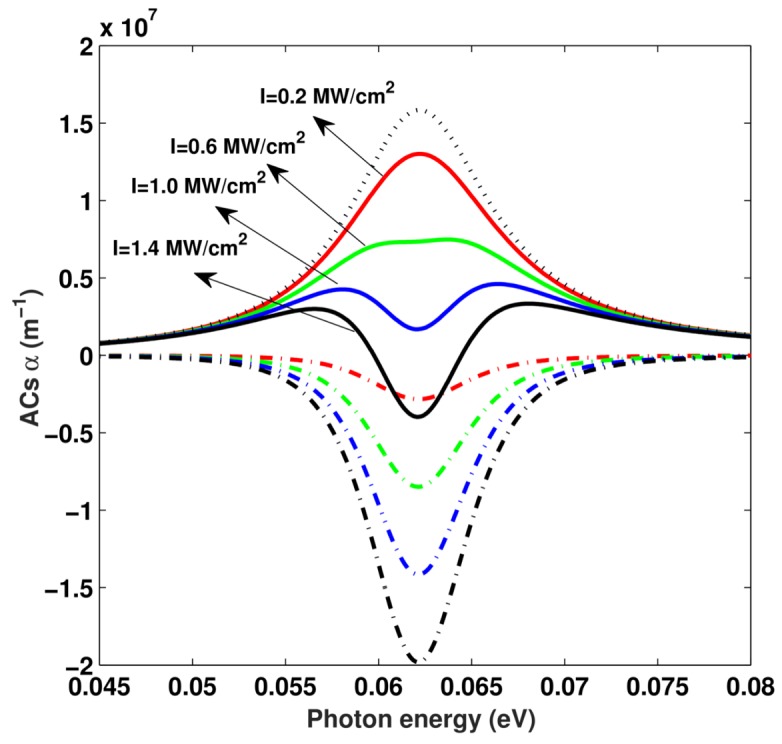
The resonance peak value of total optical absorption coefficients (OACs) as a function of incident optical intensity *I* with P=0, T=0, B=0, and L=8 nm.

To illustrate the combined effect of hydrostatic pressure *P*, temperature *T*, and applied magnetic field *B* on the total OACs α, in Figure 4, we draw the the resonance peak value of the total OACs α as a function of hydrostatic pressure *P*, temperature *T*, and applied magnetic field *B* with the incident optical intensity I=0.6 MW/cm^2^ and well width L=8 nm. As shown in Figure 4, (1) the amplitude of the resonant peaks of the total OACs α always increases with the increase in temperature *T* and and applied magnetic field *B*. However, the amplitude of the resonant peaks of the total OACs α always decreases with the increase in hydrostatic pressure *P*. (2) the effect of hydrostatic pressure *P* and applied magnetic field *B* on the total OACs are more significant than temperature *T*. (3) the increasing values of the applied magnetic field *B* causes to achieve transition from saturation absorption to unsaturation absorption (see Figure 3c). However, the transition will be easier to achieve when the incident optical intensity *I* takes a smaller value. This feature will be explained in detail in Figure 5.

Figure 5 shows the linear $\alpha^{\left(1\right)}$, third-order nonlinear $\alpha^{\left(3\right)}$, and total OACs α as a function of the photon energy *I*. Several distinct values of the incident optical intensity I=0.2,0.6,1.0,1.4 MW/cm^2^ have been taken into account with P=0,T=0,B=0. It can be seen that, in Figure 5, with the increase in incident optical intensity *I*, the magnitude of resonant peaks of the linear term $\alpha^{\left(1\right)}$ remains constant, but the magnitude of resonant peaks of third-order nonlinear term $\alpha^{\left(3\right)}$ increases. The magnitude of resonant peaks of the total OACs α is reduced by third-order nonlinear term $\alpha^{\left(3\right)}$. Therefore, the magnitude of resonant peaks of the total OACs α decreases with increasing incident optical intensity *I*. When the increasing incident optical intensity *I* is at sufficiently high intensities, the resonant peaks of the total OACs α are split into two peaks, which indicates saturation. Therefore, in the study of the optical properties of a low dimension system, the third-order nonlinear term $\alpha^{\left(3\right)}$ should be taken into account, especially when the incident optical intensity *I* is comparatively strong.

**Figure 6:**
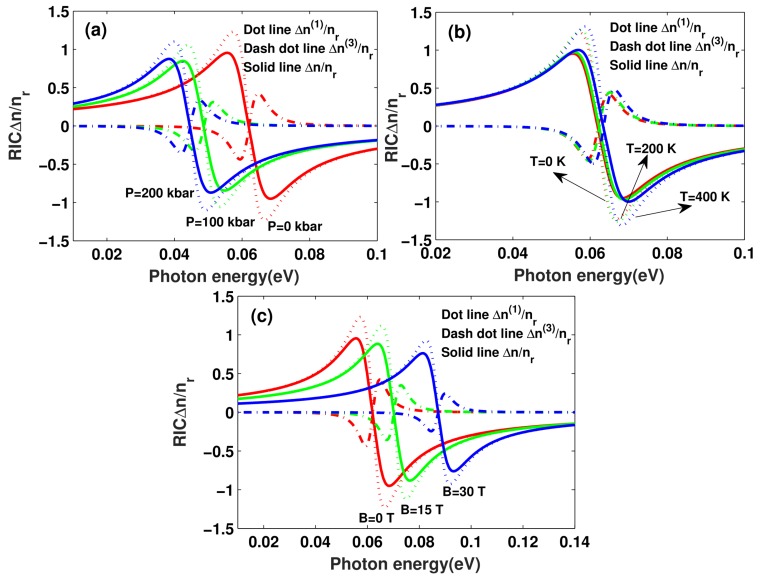
The refractive index changes (RICs) with photon energy for three different hydrostatic pressure *P* (**a**), temperature *T* (**b**), and applied magnetic field *B* (**c**) values.

In Figure 6, the linear $\Delta n^{\left(1\right)}/n_{r}$, third-order nonlinear $\Delta n^{\left(3\right)}/n_{r}$, and total RICs $\Delta n/n_{r}$ in a morse QW as functions of the incident photon energy are shown. The used parameters are same as Figure 3. From Figure 6a, it can be observed that the magnitude of resonant peaks of the linear $\Delta n^{\left(1\right)}/n_{r}$, third-order nonlinear $\Delta n^{\left(3\right)}/n_{r}$, and total RICs $\Delta n/n_{r}$ have significant enhancement with the hydrostatic pressure *P* increasing. Additionally, one readily notices the red-shift of the resonant peaks with increasing hydrostatic pressure *P*. Moreover, for larger values of the hydrostatic pressure *P*, the magnitude of resonant peaks of RICs has little change. From Figure 6b, it can be easily seen that, as the temperature *T* increases, the magnitude of resonant peaks of RICs move to the high-energy side, which shows a confinement-strength-induced blue shift. These results are consistent with the change in OACs (see Figure 3). From Figure 6c, we can see that the magnitude of resonant peaks of RICs increases with the decrease in applied magnetic field *B*. The resonant peaks of RICs will move to the right side of the curves, which predicts a strong-confinement-induced blue shift in low-dimension QWs. These features make QWs very promising candidates for NLO materials.

## 4. Conclusions

In the present paper, we investigated nonlinear OACs and RICs in GaAs/Ga_0.7_Al_0.3_As morse QWs in terms of applied magnetic field, hydrostatic pressure, and temperature. We obtained the energy eigenvalues and the energy eigenfunctions by the finite different method. Our results show that the magnitude of resonant peaks of nonlinear OACs and RICs can be controlled by these parameters. Numerical results reveal that the resonant peaks of nonlinear OACs and RICs can induce a blue- or red-shift effect by adjusting the hydrostatic pressure, applied magnetic field, or temperature. Additionally, the magnitude of resonant peaks of nonlinear OACs and RICs varies with the same symbol. In addition, it is well known that the incident optical intensity on the corresponding optical properties of a QW is very important, so it would be very useful to investigate it. A morse potential profile can be synthesized in a piecewise linear manner by using both compositional grading and layer tickness variations, such as ${\mathrm{Ga}}_{x}{\mathrm{Al}}_{1-x}\mathrm{As}$ [20]. We believe that the control of the intensity-dependent optical processes in morse QWs in terms of applied magnetic field, hydrostatic pressure, and temperature may greatly contribute to experimental studies and the practical exploitation of the quantum-size effect in devices.
